# Update: Providing Quality Family Planning Services — Recommendations from CDC and the U.S. Office of Population Affairs, 2017

**DOI:** 10.15585/mmwr.mm6650a4

**Published:** 2017-12-22

**Authors:** Loretta Gavin, Karen Pazol, Katherine Ahrens

**Affiliations:** ^1^Office of Population Affairs, U.S. Department of Health and Human Services, Rockville, Maryland; ^2^Division of Reproductive Health, CDC.

In April 2014, CDC published “Providing Quality Family Planning Services: Recommendations of CDC and the U.S. Office of Population Affairs” (QFP), which describes the scope of services that should be offered in a family planning visit and how to provide those services (e.g., periodicity of screening, which persons are in need of services, etc.) (*1*). The sections in QFP include the following: Determining the Client’s Need for Services; Contraceptive Services; Pregnancy Testing and Counseling; Clients Who Want to Become Pregnant; Basic Infertility Services; Preconception Health Services; Sexually Transmitted Disease Services; and Related Preventive Health Services. In addition, the QFP includes an appendix entitled Screening Services for Which Evidence Does Not Support Screening.

CDC and the Office of Population Affairs developed QFP recommendations by conducting an extensive review of published evidence, seeking expert opinion, and synthesizing existing clinical recommendations from CDC, agencies such as the U.S. Preventive Services Task Force (USPSTF), and professional medical associations such as the American College of Obstetricians and Gynecologists and the American Academy of Pediatrics.

The scope of preventive services related to reproductive health is constantly evolving as new scientific findings are published and clinical recommendations are modified accordingly. Being knowledgeable about the most current recommendations is an important step toward providing the highest quality care to patients. To keep QFP current with the latest recommendations, CDC and the Office of Population Affairs publish occasional updates that summarize newly published clinical recommendations. The first of these updates was published in March 2016 (*2*), and covered guidelines published during April 2014–December 2015. This report summarizes recommendations from guidelines published during January 2016–April 2017. CDC and the Office of Population Affairs prepared these updates by searching for materials from CDC, USPSTF, and other professional medical organizations that had recommendations referenced in the original QFP. When updated recommendations were identified, they were evaluated for changes in implications for providing family planning care. CDC and the Office of Population Affairs determined that none of the newly published recommendations marked a substantial shift in how family planning care should be provided, and therefore did not seek additional review to consider the implications for the QFP for this update. Technical reviews from clinical experts representing a broad range of family planning providers might be appropriate for future updates.

Updated recommendations that have implications for clinical practice for family planning providers are highlighted (Box). In addition, an updated reference list for each section in the QFP is provided for all recommendations published during January 2016–April 2017, including those that did not result in any change in recommended clinical practices for family planning providers.

BOX **Updated recommendations that might have implications for clinical practice, by section heading — Providing Quality Family Planning Services: Recommendations from CDC and the U.S. Office of Population Affairs, 2017**

**Contraceptive Services**

**Medical eligibility for contraceptive use**

The 2016 CDC recommendations update earlier 2010 recommendations for the use of specific contraceptive methods by women and men who have certain characteristics or medical conditions.

The 2016 updated recommendations include the following:

- Addition of recommendations for women with cystic fibrosis, women with multiple sclerosis, and women receiving certain psychotropic drugs or taking St. John’s wort.
- Revisions to the recommendations for emergency contraception, including the addition of ulipristal acetate (UPA) for emergency contraception.
- Revisions to the recommendations for postpartum women; women who are breastfeeding; women with known dyslipidemias, migraine headaches, superficial venous disease, gestational trophoblastic disease, sexually transmitted diseases (STDs), and human immunodeficiency virus (HIV) infection; and women who are receiving antiretroviral therapy.
- For all 2016 updated recommendations, see Tables A1 and A2: https://www.cdc.gov/mmwr/volumes/65/rr/rr6503a1_appendix.htm

**Source:** Curtis KM, Tepper NK, Jatlaoui TC, et al. U.S. medical eligibility criteria for contraceptive use, 2016. MMWR Recomm Rep 2016;65(No. RR-3).

**Selected practice recommendations for contraceptive use**

The 2016 CDC recommendations update earlier 2013 recommendations that address a select group of common, yet sometimes complex, issues regarding initiation and use of specific contraceptive methods.

Recommendations have been updated regarding when to start regular contraception after UPA emergency contraceptive pills:

- Advise the woman to start or resume hormonal contraception no sooner than 5 days after use of UPA, and provide or prescribe the regular contraceptive method as needed. For methods requiring a visit to a health care provider, such as depo-medroxyprogesterone acetate (DMPA), implants, and intrauterine devices (IUDs), starting the method at the time of UPA use may be considered; the risk that the regular contraceptive method might decrease the effectiveness of UPA must be weighed against the risk of not starting a regular hormonal contraceptive method.
  - The woman needs to abstain from sexual intercourse or use barrier contraception for the next 7 days after starting or resuming regular contraception or until her next menses, whichever comes first.
  - Any nonhormonal contraceptive method can be started immediately after the use of UPA.
  - The woman should be advised to have a pregnancy test if she does not have a withdrawal bleed within 3 weeks.

New recommendations have been made regarding medications used to ease IUD insertion:

- Misoprostol is not recommended for routine use before IUD insertion. Misoprostol might be helpful in select circumstances (e.g., in women with a recent failed insertion).
- Paracervical block with lidocaine might reduce patient pain during IUD insertion.

**Source:** Curtis KM, Jatlaoui TC, Tepper NK, et al. U.S. selected practice recommendations for contraceptive use, 2016. MMWR Recomm Rep 2016;65(No. RR-4).

**Preconception Health Services**

**Depression**

- The 2016 USPSTF recommendation for adults reaffirms the 2009 recommendation to screen all adults when staff-assisted depression care supports are in place. This replaces the 2009 recommendation regarding selective screening of adults.
- The 2016 USPSTF recommendation for adolescents aged 12–18 years reaffirms the 2009 recommendation to screen for major depressive disorder when systems are in place to ensure accurate diagnosis, effective treatment, and follow-up. The 2016 statement removes the recommendation of specific psychotherapies in recognition of decreased concern over the harms of pharmacotherapy in adolescents as long as they are adequately monitored.

**Sources:**

US Preventive Services Task Force. Screening for depression in adults. Rockville, MD: US Department of Health and Human Services, Agency for Healthcare Research and Quality; 2016.

US Preventive Services Task Force. Screening for depression in children and adolescents. Rockville, MD: US Department of Health and Human Services, Agency for Healthcare Research and Quality; 2016.

## Updated Reference List, by QFP Section

### Determining the Client’s Need for Services

American College of Obstetricians and Gynecologists’ Committee on Health Care for Underserved Women. Committee opinion no. 654: reproductive life planning to reduce unintended pregnancy.
Obstet Gynecol
2016;127:e66–9. 10.1097/AOG.000000000000131426942389

### Contraceptive Services

Curtis
KM, Tepper
NK, Jatlaoui
TC, 
U.S. medical eligibility criteria for contraceptive use, 2016.
MMWR Recomm Rep
2016;65(No. RR-3):1–103. 10.15585/mmwr.rr6503a127467196Curtis
KM, Jatlaoui
TC, Tepper
NK, 
U.S. selected practice recommendations for contraceptive use, 2016.
MMWR Recomm Rep
2016;65(No. RR-4):1–66. 10.15585/mmwr.rr6504a127467319American College of Obstetricians and Gynecologists’ Committee on Obstetric Practice. Committee opinion no. 670: immediate postpartum long-acting reversible contraception.
Obstet Gynecol
2016;128:e32–7. 10.1097/AOG.000000000000158727454734American College of Obstetricians and Gynecologists’ Committee on Gynecologic Practice Long-Acting Reversible Contraceptive Expert Work Group. Committee opinion no. 672: clinical challenges of long-acting reversible contraceptive methods.
Obstet Gynecol
2016;128:e69–77. 10.1097/AOG.000000000000164427548557

### Clients Who Want to Become Pregnant

Pfeifer
S, Butts
S, Fossum
G, ; Practice Committee of the American Society for Reproductive Medicine in collaboration with the Society for Reproductive Endocrinology and Infertility. Optimizing natural fertility: a committee opinion.
Fertil Steril
2017;107:52–8. 10.1016/j.fertnstert.2016.09.02928228319

### Preconception Health Services

American College of Obstetricians and Gynecologists’ Committee on Health Care for Underserved Women. Committee opinion no. 654: reproductive life planning to reduce unintended pregnancy.
Obstet Gynecol
2016;127:e66–9. 10.1097/AOG.000000000000131426942389Kim
DK, Riley
LE, Harriman
KH, Hunter
P, Bridges
CB. Advisory Committee on Immunization Practices recommended immunization schedule for adults aged 19 years or older—United States, 2017.
MMWR Morb Mortal Wkly Rep
2017;66:136–8. 10.15585/mmwr.mm6605e228182599PMC5657965Robinson
CL, Romero
JR, Kempe
A, Pellegrini
C; Advisory Committee on Immunization Practices (ACIP) Child/Adolescent Immunization Work Group. Advisory Committee on Immunization Practices recommended immunization schedule for children and adolescents aged 18 years or younger—United States, 2017.
MMWR Morb Mortal Wkly Rep
2017;66:134–5. 10.15585/mmwr.mm6605e128182607PMC5657960US Preventive Services Task Force. Folic acid supplementation for the prevention of neural tube defects: preventive medication. Rockville, MD: US Department of Health and Human Services, Agency for Healthcare Research and Quality; 2017. https://www.uspreventiveservicestaskforce.org/Page/Document/UpdateSummaryFinal/folic-acid-for-the-prevention-of-neural-tube-defects-preventive-medicationUS Preventive Services Task Force. Depression in adults: screening. Rockville, MD: US Department of Health and Human Services, Agency for Healthcare Research and Quality; 2016. https://www.uspreventiveservicestaskforce.org/Page/Document/UpdateSummaryFinal/depression-in-adults-screening1US Preventive Services Task Force. Depression in children and adolescents: screening. Rockville, MD: US Department of Health and Human Services, Agency for Healthcare Research and Quality; 2016. https://www.uspreventiveservicestaskforce.org/Page/Document/UpdateSummaryFinal/depression-in-children-and-adolescents-screening1

### Sexually Transmitted Disease Services

US Preventive Services Task Force. Syphilis infection in nonpregnant adults and adolescents: screening. Rockville, MD: US Department of Health and Human Services, Agency for Healthcare Research and Quality; 2016. https://www.uspreventiveservicestaskforce.org/Page/Document/UpdateSummaryFinal/syphilis-infection-in-nonpregnant-adults-and-adolescents

### Related Preventive Health Services

US Preventive Services Task Force. Breast cancer: screening. Rockville, MD: US Department of Health and Human Services, Agency for Healthcare Research and Quality; 2016. https://www.uspreventiveservicestaskforce.org/Page/Document/UpdateSummaryFinal/breast-cancer-screening1US Preventive Services Task Force. Gynecological conditions: periodic screening with the pelvic examination. Rockville, MD: US Department of Health and Human Services, Agency for Healthcare Research and Quality; 2017. https://www.uspreventiveservicestaskforce.org/Page/Document/UpdateSummaryFinal/gynecological-conditions-screening-with-the-pelvic-examination

### Screening Services for Which Evidence Does Not Support Screening

US Preventive Services Task Force. Genital herpes infection: serologic screening. Rockville, MD: US Department of Health and Human Services, Agency for Healthcare Research and Quality; 2016. https://www.uspreventiveservicestaskforce.org/Page/Document/UpdateSummaryFinal/genital-herpes-screening1

References1. Gavin
L, Moskosky
S, Carter
M, . Providing quality family planning services: recommendations of CDC and the U.S. Office of Population Affairs.
MMWR Recomm Rep
2014;63(No. RR-04).247596902. Gavin
L, Pazol
K. Update: providing quality family planning services—recommendations from CDC and the U.S. Office of Population Affairs, 2015.
MMWR Morb Mortal Wkly Rep
2016;65:231–4. 10.15585/mmwr.mm6509a326963363
